# The Effectiveness and Cost-Effectiveness of Screening and Brief Alcohol Intervention to Reduce Alcohol Consumption in Young People in the High School Setting: A Pragmatic Randomized Controlled Trial (SIPS JR-HIGH)

**DOI:** 10.1093/alcalc/agab087

**Published:** 2022-02-03

**Authors:** Simon Coulton, Emma L Giles, Grant J McGeechan, Paolo Deluca, Colin Drummond, Denise Howel, Eileen Kaner, Elaine McColl, Ruth McGovern, Stephanie Scott, Harry Sumnall, Luke Vale, Viviana Albani, Sadie Boniface, Jennifer Ferguson, Eilish Gilvarry, Nadine Hendrie, Nicola Howe, Amy Ramsay, Dorothy Newbury-Birch

**Affiliations:** Centre for Health Service Studies, University of Kent, Canterbury, UK; School of Health & Life Sciences, Teesside University, Middlesbrough, UK; Centre for Applied Psychological Science, Teesside University, Middlesbrough, UK; Institute of Psychiatry, Psychology & Neuroscience, King’s College, London, UK; Institute of Psychiatry, Psychology & Neuroscience, King’s College, London, UK; Population Health Sciences Institute, Newcastle University, Newcastle-upon-Tyne, UK; Population Health Sciences Institute, Newcastle University, Newcastle-upon-Tyne, UK; Population Health Sciences Institute, Newcastle University, Newcastle-upon-Tyne, UK; Population Health Sciences Institute, Newcastle University, Newcastle-upon-Tyne, UK; Population Health Sciences Institute, Newcastle University, Newcastle-upon-Tyne, UK; Faculty of Health, Liverpool John Moores University, Liverpool, UK; Population Health Sciences Institute, Newcastle University, Newcastle-upon-Tyne, UK; Population Health Sciences Institute, Newcastle University, Newcastle-upon-Tyne, UK; Institute of Alcohol Studies, London, UK; School of Social Sciences, Humanities & Law, Teesside University, Middlesbrough, UK; Northumberland, Tyne & Wear NHS Foundation Trust, St Nicholas Hospital, Newcastle-upon-Tyne, UK; Centre for Health Service Studies, University of Kent, Canterbury, UK; Newcastle Clinical Trials Unit, Newcastle University, Newcastle-upon-Tyne, UK; Institute of Psychiatry, Psychology & Neuroscience, King’s College, London, UK; School of Social Sciences, Humanities & Law, Teesside University, Middlesbrough, UK

## INTRODUCTION

The Chief Medical Officer for England recommends that young people remain alcohol free until 18 years of age. This recommendation was accompanied by advice that young people under the age of 15 should abstain completely, but if those aged 15 to 17 years choose to consume alcohol, they should drink no more than once per week under adult supervision and the weekly quantity consumed should not exceed the daily adult daily limits of six units (Donaldson, 2009).

In the UK alcohol consumption is on decline among adolescents, although those who do drink tend to drink more (Emerson et al., 2016). When compared with other Western European countries, the UK has some of the highest levels of drinking among adolescents and the North East England has one of the highest levels of adolescent alcohol consumption in the UK (NHS Digital, 2016a), with 49% of 11 to 15 year olds indicating that they have consumed alcohol (Fuller, 2015). The proportion of young people who consume alcohol in the UK increases with age; in 2018, 11% of females and 9% of males aged 11–15 years reported consuming alcohol in the past week (NHS Digital, 2018). At the age of 11 years, 2% of adolescents report consuming alcohol in the past week and this rises to 23% by the age of 15 (NHS Digital, 2018). The mean weekly alcohol consumed is lowest among 11- to 13-year-olds at 8.8 UK units, where one unit equates to 8 g of ethanol, and the highest is among 15-year-olds at 11.1 units. Males consume more alcohol on average than females, 11.1 versus 9.6 units.

The British Birth Cohort Study followed up 16,000 births born between 5 and 11 April 1970 at ages 5, 10, 16 and 30 years. Data from this study were used to explore the relationship between alcohol use during adolescence and negative consequences in adulthood (Viner and Taylor, 2007). More frequent heavy episodic alcohol use was associated with higher rates of alcohol dependence, homelessness, lower educational attainment and greater involvement with the criminal justice system. More proximal consequences of adolescent alcohol use include increased risk of injury, higher prevalence of anxiety and depression, more regretted and unsafe sexual activity, worse peer and family relationships and an increased likelihood of being a victim of crime (Newbury-Birch et al., 2009). Alcohol use in adolescence is also associated with an increased prevalence of smoking, poorer quality of life and greater levels of emotional dysregulation, conduct disorder and hyperactivity (Donoghue et al., 2017). Alcohol and substance use are the most common reason adolescents are excluded from education in the UK (Department for Education, 2017) and the number of alcohol-related exclusions have risen by 57% in the past 5 years.

Alcohol Screening and Brief Intervention (ASBI) is a form of secondary prevention that targets a population who are already consuming alcohol at a level that may be risking their current or future health. This approach has become the cornerstone of alcohol treatment for at-risk alcohol users (Babor and Higgins-Biddle, 2001; Milner and Rollnick, 2013; NHS Digital, 2016b). They are typically delivered to opportunistically identified, non-treatment-seeking populations by generalist, rather than addiction specialist, practitioners in a variety of settings. They largely consist of two differing approaches. First, simple structured advice following screening that seeks to raise awareness of alcohol use through the provision of personalized feedback and simple practical steps that may be employed to reduce drinking. Second, extended brief interventions, usually involving more intensive behavioural change counselling, whereby individuals are given the opportunity to explore their alcohol use as well as their motivations and strategies to effect change (National Institute for Health and Social Care Excellence, 2010). Both approaches to brief intervention share a common goal of helping people to reduce alcohol consumption, aiming for moderation rather than abstinence and to promote better physical and psychological health. While there is a wide variation in the duration and frequency of brief interventions they are usually delivered as a single session or a series of related sessions, not exceeding five, and last between 5 and 60 min (Kaner et al., 2018).

There is a paucity of research exploring the secondary prevention targeting alcohol users in the school setting; what evidence there is tends to focus on older adolescents and young adults in college and university settings (National Institute for Clinical and Health Excellence, 2010). Most of the research addressing younger adolescents in school settings has employed a primary prevention approach, which aims to prevent the onset of unhealthy alcohol use by targeting all young people irrespective of whether they drink or not. This body of research has typically focussed on universal interventions comprising of classroom curricula, parents and family-based interventions or a combination of the two. One large Randomised Controlled Trial (RCT) of a universal classroom intervention delivered in the UK was the Steps Towards Alcohol Misuse Prevention Programme trial (STAMPP; Sumnall et al., 2017), which investigated the effectiveness of a combined school and parent intervention. The study found a significant reduction in the frequency of heavy episodic alcohol use among 12- to 13-year-olds at 33-month follow-up, although the effects had diminished by 57 months. Notably, the intervention was delivered at class level and targeted the whole class rather than individuals who exhibited risky drinking behaviour.

Two pilot studies have been conducted of ASBI in the school setting. One conducted in Mexico (Martínez Martínez et al., 2008) targeted 40 moderate-to-high-risk drinkers, mainly male (65%), with an average age of 16 years. The ASBI group received one 90-min ASBI compared with a waiting list control. At the follow-up points of 3 and 6 months, the ASBI group showed a significant reduction in the amount of alcohol consumed compared with the control. The second study conducted in the USA targeted 79 young people who had a substance use disorder (Winters and Leitten, 2007). Most participants were male (52%), with an average age of 16 years. The ASBI comprised of two 60-min sessions, with one group also receiving a parental session. Significant reductions were reported for the number of days alcohol was consumed compared with the assessment-only control group. These two school-based studies therefore suggest the potential effectiveness of using a school setting to deliver ASBI to young people.

A meta-analysis of school-based interventions to reduce risk taking behaviours suggested that interventions in school settings may be effective in reducing alcohol use (Wilson et al., 2001), whereas a more recent review exploring school-based interventions to reduce multiple risk behaviours demonstrated only a small effect on alcohol consumption (Bonnell et al., 2013). Overall, there is mixed evidence of whether school-based ASBI can be beneficial (Hale et al., 2014) and very limited evidence of the effect for high risk drinkers (Gmel et al., 2012). Similarly, there is literature indicating the potential benefits of family- and school-based interventions in reducing alcohol use (Toumbourou et al., 2013) but the evidence is from outside the UK education system and the evidence from the UK does not explore the use of targeted ASBI.

## METHODS

Prior to the embarking on the reported study, we conducted a small pilot cluster randomized controlled trial to explore the acceptability and feasibility of ASBI delivered in the school setting (O’Neil et al., 2012; Newbury-Birch et al., 2014) and to inform the design of this trial. Young people, aged 14–15 years, who indicated frequent heavy episodic alcohol use and consented to take part (*n* = 229), were allocated to one of the three arms; a control arm of a simple advice leaflet, a 30-min brief intervention consisting of structured advice delivered by school pastoral staff and an advice leaflet (intervention 1); the same brief intervention and advice leaflet augmented with a 60-min intervention including parents and caregivers (intervention 2). A total of 202 (88%) participants were followed up at 12 months. While this pilot study confirmed the proposed research procedures were feasible and acceptable to young people and schools, with high rates of engagement for control and intervention 1, there were poor levels of engagement with intervention 2 from parents and caregivers and so it was dropped from the main trial.

### Design

A multi-centre, prospective, pragmatic, two-arm, individually randomized controlled trial was conducted in accordance with the declaration of Helsinki, and ethical approval was granted by the Teesside University Ethics Committee (ref 164/15). The trial was registered (ref ISRCTN45691494). A full protocol was published in advance of analysis of the trial data (Giles et al., 2016).

### Participants

Adolescents aged 14–15 years, in high schools located in four areas of England (North East, North West, Kent and London), were eligible for inclusion if they had not been opted out of the study by parents, screened positive on the Adolescent-Single Alcohol Question (A-SAQ; Williams and Vinson, 2001; Canagasaby and Vinson, 2005) and were willing and able to provide informed consent for trial participation. We excluded participants who were already seeking help for an alcohol use disorder or had a recognized mental health condition or presented with challenging behaviour as identified by school staff.

### Sample size calculation

We used estimates from our pilot study (Newbury-Birch et al., 2014) to estimate likely school size, eligibility and consent rates and aimed to detect a small standardized effect size difference in alcohol consumed in the previous 28-days at 12 months of 0.3, equating to a ratio of 1.5 in geometric means. With power at 80%, an alpha of 0.05, a two-sided test required follow-up data from 176 students in each arm at 12 months, a total of 352. Our evidence suggested loss to follow-up at 12 month was unlikely to exceed 20%, so the numbers needed to recruit in each arm were inflated to 220, giving a total sample required of 440.

### Randomization

Eligible and consenting participants were randomized with equal probability of being allocated to intervention or control. Allocation was operationalized using opaque, sequentially numbered sealed envelopes with the allocation only being revealed after consent had been obtained and the baseline assessment conducted. The allocation schedule was designed independent of the research team and employed random permuted blocks of variable length stratified by school.

## PROCEDURE

Prior to conducting screening and eligibility assessments, parents or caregivers of all potentially eligible participants were able to opt out their children from the trial. By not opting out it was assumed parents or caregivers were happy for their child to engage in the screening and if they were screened positive, and provided assent, participate in the trial. All the young people in the year group, who were not opted out by their parents, viewed a bespoke video animation containing information on the trial and expectations for participants. Screening and baseline assessment were conducted on paper during a scheduled Personal, Health and Social Education (PHSE) or registration class. Young people were given options to not complete the assessment, complete the assessment anonymously or complete the assessment and provide their name and class. Those young people who completed the assessment, screened positive on the A-SAQ, and left their name were eligible for inclusion in the trial.

### Delivery of the intervention

#### Intervention arm

This comprised a 30-min face-to-face intervention delivered by the learning mentor or equivalent staff member with pastoral care responsibilities within the school. The essential components were developed in the feasibility trial and the format was developed in collaboration with young people. The result was an A3 sheet detailing a six-step intervention detailed in full, using TIDieR criteria in Table 1.

**Table 1:** Summary of trial arm components

Component	Control condition	Brief alcohol intervention condition
Rationale, theory or goal	Comparison condition	Motivational interview to reduce alcohol use
Materials	Healthy lifestyle leaflet	Alcohol advice leaflet
Procedure	Provision of healthy lifestyle leaflet by learning mentor in school.	Feedback on alcohol screening results, advice on recommended alcohol consumption levels and comparison with participants alcohol consumption. Raising awareness of risks associated with excessive alcohol consumption and delivery of behavioural change counselling.
Intervention provider	Learning mentor	Learning mentor
Delivery mode	Information leaflet	Face-to-face discussion and information leaflet
Location	School	School
Session duration and frequency	1 min	Up to 30 min
Tailoring	None	Yes
Fidelity assessment	All sessions audio recorded and a random 20% checked by an experienced alcohol counsellor to explore differentiation from the intervention condition in terms of the advice provided.	All sessions audio recorded and assessed for fidelity using the Behaviour Change Counselling Index (BECCI) by an experienced alcohol counsellor.
Fidelity outcome	All sessions assessed were considered appropriately differentiated.	Mean BECCI score was 1.6 indicating behaviour change counselling was being delivered.

In brief, the intervention consisted of feedback of screening results and raising awareness of how many units of alcohol were contained in commonly consumed drinks, an exploration of a typical drinking day to identify behaviours that may be the focus of change, exploring personally relevant risks of alcohol consumption, identifying motivational factors, exploring confidence to change, barriers and facilitators of behaviour change, developing an action and coping plan for changing drinking behaviour. In addition, pupils received PHSE as usually provided by the school.

#### Interventionist training

Learning mentors, or equivalents, were trained on school premises by an experienced ASBI trainer. Training sessions lasted 3 h and involved both theoretical and practical aspects of intervention delivery, practice and role play. Training was accompanied by a detailed intervention manual. Prior to engaging in the trial, interventionists practiced and recorded the intervention and were assessed as being competent by the lead trainer. Weekly supervision was provided to the interventionists by the research team.

#### Control arm

The control arm is detailed in Table 1. Participants in the control arm of the study received a healthy lifestyle leaflet addressing diet and exercise. No specific feedback on the alcohol screening results was provided. In addition, pupils received PHSE as usually provided by their school.

#### Hypotheses

Our primary null hypothesis was that adding ASBI in addition to PHSE for adolescents in school was no more effective than PHSE alone in reducing the quantity of alcohol consumed 12 months after randomization.

Our secondary null hypothesis was that adding ASBI in addition to PHSE for adolescents in school was no more cost-effective than PHSE alone.

### Outcome measures

#### Screening

Potential participants were screened using the A-SAQ (Williams and Vinson, 2001; Canagasaby and Vinson, 2005). This single question assesses the frequency of heavy episodic alcohol consumption, defined as six or more standard drinks in a single occasion where one standard drink equates to 8 g of ethanol, over the previous 6 months. Responses include ‘never’, ‘less than four times’, ‘four or more times but not every month’, ‘more than once a month but not every week’, ‘every week but not every day’ and ‘every day’. Endorsing ‘four or more times but not every month’ or more frequent is a positive screen.

#### Demographic

At baseline participants were asked to provide information on their sex, ethnicity and whether they had smoked tobacco in the past 30 days.

#### Primary outcome measure

The primary outcome measure, assessed at 12 months post randomization, was total alcohol consumed, in units of alcohol, in the 28-days prior to the assessment. This was assessed using the Time Line Follow Back method (TLFB; Sobell and Sobell, 1995).

#### Secondary outcome measures

Percent days abstinent in the previous 28 days at 12 months post randomization was derived from the TLFB. We also assessed participants scores on the Alcohol Use Disorders Identification Test (AUDIT; Saunders et al., 1993) and the three AUDIT consumption items (AUDIT-C) at baseline and 12-months.

Alcohol-related problems were assessed using the Rutgers Alcohol Problems Inventory (RAPI; (Shono et al., 2018)). Motives for drinking were assessed using the revised Drinking Motives Questionnaire. This measures motives for drinking over four domains: social, coping, enhancement and conformity (DMQ-R; Harbke et al., 2019). General psychological health was assessed using the Warwick–Edinburgh Mental Wellbeing Scale (WEMWBS; Clarke et al., 2011).

The primary outcome for the economic evaluation was health utility, estimated using the EQ-5D-3L questionnaire (EQ-5D-3L; EuroQol Research, 1990). This questionnaire considers five dimensions of health: mobility, self-care, usual activities, pain and discomfort and anxiety and depression, and is validated for those aged 12 years or older. The costs of delivery were based on the actual cost each item of resource, including staff time and materials, used in the training and intervention. Differences in public sector resource use between the intervention and control arms were assessed at 12 months using data collection form designed for this population derived from the client service receipt inventory tool.

### Statistical analysis

We used Stata 15 to analyse the trial by treatment allocated; this is analysing participants as members of their allocated group irrespective of the intervention received. The analytical team remained blind to participant allocation until they had completed the primary analysis.

Descriptive statistics were used to report the demographic and outcome data by trial arm at baseline and follow-up. It was planned that multiple linear regressions would be used to compare the primary outcome between trial arms at follow-up, either on the original scale or after a logarithmic transformation if the outcome data were skewed. However, the degree of zero-inflation in the primary outcome was much higher than expected, and the planned analysis was not appropriate. We explored the use of hurdle models, but convergence could not be achieved. As an alternative we employed quantile regression modelling the median number of units consumed in each group and adjusting by known baseline covariates: region, gender, level of deprivation and baseline AUDIT score. Secondary outcomes were analysed in a similar manner with the exception of the proportion who consumed changed from a higher to lower risk of alcohol consumption between baseline and month 12, from AUDIT score >3 to 3 or less (Coulton et al., 2019), this was analysed using a logistic regression model adjusting for the same covariates as used in the primary analysis. We planned to conduct a secondary analysis of the primary outcome including only those who had received the treatment as allocated, a per-protocol analysis, but as all participants received the allocated treatment this analysis was not necessary. We conducted post hoc analysis to generate Bayes factors to aid the interpretation of the findings. Bayes factors allow us to interpret the strength of support for the alternative hypothesis (Dienes et al., 2018).

We conducted a cost-effectiveness analysis, assessing both resources used and any resulting change in health utility. We used data provided by individual participants to estimate mean differences in mean costs between intervention and control, and converted their EQ-5D-3L scores at baseline and 12 months to Quality Adjusted Life Years (QALY) using the area under the curve approach. We adopted a distinct perspective that encompassed societal, health and personal social services.

As health economic data are usually subject to sampling error, we employed stochastic sensitivity analysis in the form of 1500 non-parametric bootstrapped replications of costs and effects stratified by gender, allocated group and geographical location to derive 95% confidence intervals of the incremental cost-effectiveness ratio and the cost-effectiveness acceptability curves (CEACs) showing the probability that interventions were cost-effective over a range of willingness to pay thresholds ranging between £20,000 and £30,000 per QALY in the UK.

For cost services we used local costs where available and supplemented with published national costs (Personal Social Services Research, 2015; Department of Health and Social Care, 2016) and information from previous alcohol studies (Coulton et al., 2006; Coulton et al., 2008). As all costs occurred within 12 months no discounting was applied. We estimated the cost of screening and delivering the intervention and control by estimating the actual costs of activities including the cost of training, trainers and materials.

## RESULTS

### Sample and follow-up

The recruitment of schools took place between November 2015 and June 2016. To maximize generalizability, we only excluded private schools. We approached all government-funded schools in the research areas. Over the period 154 schools were approached to participate, of which 33 agreed. The most common reason for non-participation was lacking staff or time to participate or having a specific school policy not to participate in research.

**Figure 1:**
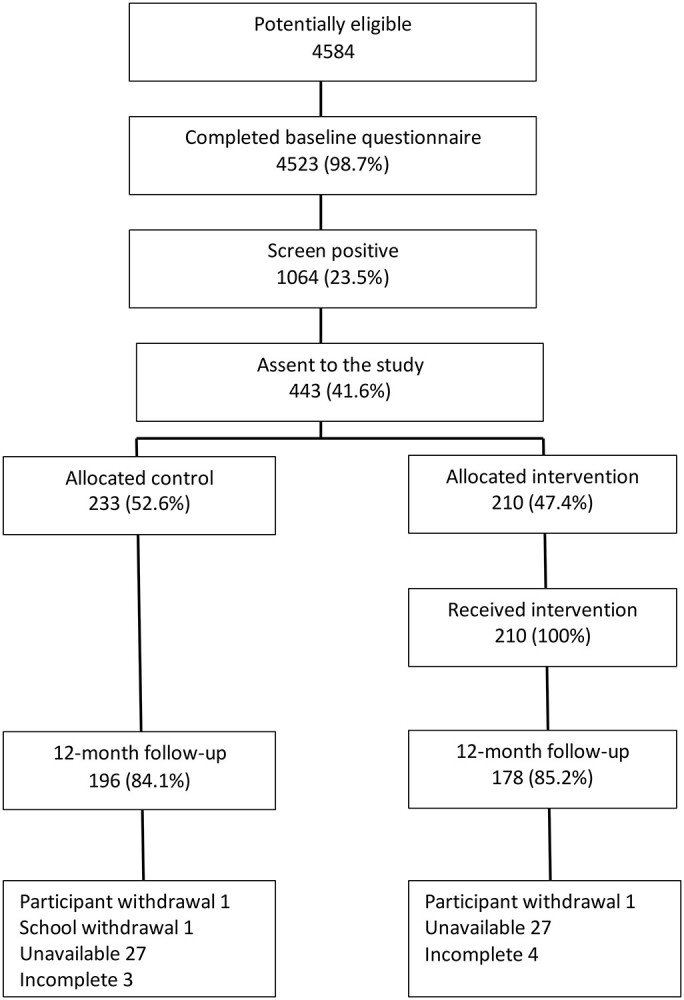
CONSORT flow diagram.

Figure 1 presents the trial CONSORT diagram indicating trial recruitment, allocation and follow-up at 12 months.

Of those identified as potentially eligible, 99% (4523) completed the screening tool and 24% (1064) responded with a positive screen, 443 (42%) assented to participate in the study meeting our sample size requirements and all of those allocated to the intervention received it. At 12 months we exceeded our target of 80% follow-up. Half of the sample were male (50.3%) and 90.2% were identified as white ethnicity; the mean AUDIT score at baseline was 7.6 (SD 5.8). Table 2 presents the baseline demographic and outcome variables by allocated group and confirms that these were similar.

**Table 2:** Baseline demographic and outcome variables by allocated group

	Intervention *n* = 210	Control *n* = 233
**Demographic variables**		
Male *n* (%)	104 (49.5)	118 (50.6)
White ethnicity *n* (%)	189 (90.0)	213 (91.4)
Smoked in the past 30 days *n* (%)	59 (28.1)	70 (30.3)
Regretted sexual intercourse *n* (%)	22 (10.5)	16 (6.9)
Unsafe sexual intercourse *n* (%)	22 (10.5)	23 (9.9)
**Outcome variables**		
Mean AUDIT score (SD)	7.6 (5.6)	7.6 (6.4)
Median AUDIT score (IQR)	6 (3; 11)	6.5 (3; 10)
Mean AUDIT-C score (SD)	3.8 (2.1)	4.0 (2.4)
Median AUDIT-C score (IQR)	3 (2; 5)	4 (2; 5)
Mean RAPI score (SD)	8.1 (9.9)	6.5 (8.7)
Median RAPI score (IQR)	5 (1; 12)	3 (1; 9)
Mean WEMWBS score (SD)	45.4 (12.0)	46.4 (11.4)
Mean DMQ-R – coping score (SD)	1.8 (1.0)	1.7 (0.9)
Mean DMQ-R – social score (SD)	2.7 (1.1)	2.5 (1.0)
Mean DMQ-R – conforming score (SD)	1.3 (0.7)	1.3 (0.7)
Mean DMQ-R – enhancement score (SD)	2.1 (1.0)	2.0 (1.0)

### Primary outcome analysis

About a quarter of young people indicated that they had consumed no alcohol in the previous 28-days at the 12-month follow-up, 21% in the intervention group and 28% in the control group. Table 3 presents the unadjusted and adjusted median differences and 95% confidence intervals for the primary outcome, and total units of alcohol consumed in the previous 28 days at 12 months. This indicates no significant differences between the groups, and the Bayes factor comparing the intervention versus control was 0.30 and reinforces the null findings of the primary outcome.

**Table 3:** Twelve-month outcomes and difference in medians favouring intervention by allocated group

	Intervention (*n* = 178)		Control (*n* = 196)		Difference in medians Intervention versus control (95% CI)	
	Mean (SD)	Median (IQR)	Mean (SD)	Median (IQR)	Unadjusted	Adjusted^1^
Units consumed in past 28 days^2^	16.2 (27.9)	7.3 (1.8; 18.5)	13.2 (17.5)	7.7 (0; 18)	−0.5 (−4.2; 3.1)^3^	0.8 (−2.4; 4.0)^3^
Percent days abstinent in past 28 days	92.1 (9.1)	92.9 (89.3; 96.4)	93 (7.4)	96.4 (89.3; 100)	−3.6 (−4.9; −2.2)^3^	−0.4 (−2.2; 1.5)^3^
Drinks per drinking day in past 28 days^2^	5.3 (5.2)	4.2 (1.5; 7.8)	4.9 (5.2)	3.9 (0; 7.6)	0 (−1.3; 1.3)^3^	−0.5 (−1.6; 0.6)^3^
AUDIT score	5.7 (4.2)	5 (3;8)	5.5 (4.3)	5 (2; 8)	0 (−1.1; 1.1)	−0.1 (−1.0; 0.8)
AUDIT-C score	3.7 (2.1)	3 (2; 5)	3.4 (2.2)	3 (2; 5)	0 (−0.6; 0.6)	0.1 (−0.4; 0.7)
RAPI	4.5 (5.3)	3 (0; 7)	4.0 (4.8)	3 (0; 6)	0 (−1.3; 1.3)	0.2 (−0.8; 1.2)
WEMWBS	48.9 (9.0)	50 (43; 55)	48.6 (9.4)	49 (41; 55)	1.0 (−1.6; 3.6)	1.7 (−0.7; 4.1)
DMQ-R – Coping	1.5 (0.6)	1.4 (1; 1.8)	1.6 (0.7)	1.4 (1; 2)	0 (−0.2; 0.2)	−0.1 (−0.3; 0.1)
DMQ-R – Social	2.7 (1.0)	2.6 (2; 3.6)	2.6 (1.0)	2.4 (1.8; 3.2)	0.2 (−0.1; 0.5)	0.1 (−0.2; 0.5)
DMQ-R – Conforming	1.1 (0.4)	1 (1; 1.2)	1.1 (0.3)	1 (1; 1.2)	0 (−0.2; 0.2)	0 (−0.04; 0.04)
DMQ-R – Enhancement	1.9 (0.9)	1.6 (1.2; 2.4)	1.9 (0.8)	1.8 (1.2; 1.8)	−0.2 (−0.4; 0.03)	−0.1 (−0.3; 0.2)

^1^Adjusted for covariates in the model; baseline value where available, gender, index of deprivation and baseline AUDIT score.

^2^UK standard unit; 8 g or 10 ml of ethanol.

^3^Difference in medians derived using quantile regression.

### Secondary outcome analysis

Adjusted mean differences for secondary outcomes are presented in Table 3. At 12-months 60% of those in the intervention arm and 59% of those in the control arm stated that they had reduced the amount of alcohol they consumed. No evidence of differences was found between the intervention and control groups on any secondary outcomes. Logistic regression analysis of those who reduced consumption, from higher to lower risk between baseline and 12 months showed no evidence of association with trial arm with an adjusted odds ratio of 1.04 (95% CI 0.53 to 1.56) with the control group as the referent category.

### Economic analysis

The marginal additional mean cost of delivering the intervention versus the control was £31.30 (95% CI 30.9 to 31.7) per intervention participants. The intervention group had higher mean costs on average over the 12-month follow-up than the control group, £79, although the confidence interval included zero (95% CI -£104 to £260). The difference in mean QALY’s was −0.004 (95% CI -0.019 to 0.011) but again the confidence interval included zero.

The CEAC indicates that there is only a 20% probability that the intervention is cost-effective at a willingness to pay threshold of £20,000 to £30,000. Sensitivity analysis was conducted where extreme values for use of GP, nurse and social worker values were truncated. Doing this made no difference to the overall findings. In addition, we explored the influence of missing data by conducting a sensitivity analysis including values of costs and QALY derived from multiple imputation. Again, this had no influence on the findings.

## DISCUSSION

The overall aim of the study was to conduct a definitive, appropriately powered pragmatic randomized controlled trial to evaluate the effectiveness and cost-effectiveness of ASBI for higher risk adolescent alcohol users in a school setting. We achieved both our recruitment and retention targets and we found no significant effects of the intervention when compared with the control. The calculation of posterior Bayes Factors supported the finding that ASBI was no more effective than screening in addition to PHSE provided as usual by the school.

Our economic analysis highlighted that there was only a 20% likelihood that the intervention was cost-effective compared with the control. This finding did not markedly change in all the sensitivity analyses conducted.

These findings appear to contrast with previous studies of brief interventions delivered in school settings (Winters and Leitten, 2007; Martínez Martínez et al., 2008), in part because these studies tended to be single-site, small-scale and underpowered and consequently are prone to type I error, incorrectly rejecting the null hypothesis. It is also of note that positive evidence of the efficacy of ASBIs has not translated into evidence of effectiveness when evaluated using large-scale, multi-centre, pragmatic trial designs. This finding has important implications because pragmatic trials evaluate interventions in real-world environments rather than ideal, tightly controlled environments.

Our results concur with a number of more recent studies of ASBI in adolescent populations (Deluca et al., 2020; Deluca et al., 2021), school settings (Strom et al., 2014) and adult populations (Kaner et al., 2013; Drummond et al., 2014) that indicate that ASBI is not any more effective than screening and simple advice alone.

It should also be noted that most young people indicated they had reduced their alcohol consumption at 12 months compared with baseline, and this occurred equally in both arms of the study. This is likely to be an artefact of the trial design, regression to the mean, whereby when participants are selected because they consume alcohol above a threshold, they tend to fall towards the population mean over time.

Primary prevention approaches delivered in schools take a variety of forms but tend to focus on education about risks and the development of life skills. Two systematic reviews have found a paucity of evidence for other forms of primary prevention delivered in school settings but have highlighted the emerging evidence for the ‘unplugged’ program (Foxcroft and Tsertsvadze, 2011; Agabio et al., 2015). This program involves teacher-based delivery of a multi-session educational intervention over 12 weeks that addresses alcohol and other substance use and explores knowledge and attitudes in addition to both inter- and intra- personal skill development. Participation in the program is associated with both short- and long-term significant reductions in the frequency of heavy episodic alcohol use and alcohol-related problems. In a similar vein, the STAMPP intervention is a universally delivered primary prevention program targeting both 12–13 years olds in school and their parents; this intervention approach demonstrated significant differences in heavy episodic alcohol use at 33 months, although no differences in alcohol-related problems (Sumnall et al., 2017).

Limitations of the study include the fact that only a minority of schools approached were willing to participate ( 21%). Of those who were not willing to participate, the most common reasons were a lack of time and resources, although a number cited that they did not consider alcohol use something that should be addressed within the school environment. Those that did participate are likely to be representative of schools who would deliver an alcohol intervention of this sort if available. An additional limitation relates to our use of self-report drinking as the primary outcome and the potential accuracy of this approach highlighted in other studies, particularly as it relates to potential recall bias (Percy et al., 2005; Shillington et al., 2011). However adolescent self-report of alcohol use is generally considered to be reliable (Leigh et al., 1998; Lintonen et al., 2004) and as the study was individually randomized within schools any bias would be equally distributed between the intervention and control groups.

The reported study is an evaluation of ASBI in real-life environments. It included a large sample size, appropriate methodology and the use of valid and reliable outcome measures. While there is some evidence for effectiveness, universally delivered primary prevention approaches for this population, the combined effectiveness and cost-effectiveness analysis of this study suggest it is not worthwhile implementing ASBI as a secondary prevention approach for adolescents in the school setting.

## Supplementary Material

Coulton040721_CONSORT_agab087

## TRIAL REGISTRATION

Current Control Trials ISRCTN45691494.

## References

[ref1] Agabio R, Trincas G, Floris F, et al. (2015) A systematic review of school-based alcohol and other drug prevention programs. Clin Pract Epidemiol Ment Health 11:102–12. 25834630 10.2174/1745017901511010102 PMC4378029

[ref2] Babor TF, Higgins-Biddle J. (2001) Brief Intervention for Hazardous and Harmful Drinking: A Manual for Use in Primary Care. Geneva World Health Organization, Department of Mental Health and Substance Dependence.

[ref3] Bonnell C, Jamal F, Harden A, et al. (2013) Systematic review of the effects of schools and school environment interventions on health: evidence mapping and synthesis. Public Health Res 1:1–320. 25642578

[ref4] Canagasaby A, Vinson DC. (2005) Screening for hazardous or harmful drinking using one or two quantity-frequency questions. Alcohol Alcohol 40:208–13. 15797883 10.1093/alcalc/agh156

[ref5] Clarke A, Friede T, Putz R, et al. (2011) Warwick-Edinburgh mental well-being scale (WEMWBS): validated for teenage school students in England and Scotland. A mixed methods assessment. BMC Public Health 11:487. 21693055 10.1186/1471-2458-11-487 PMC3141456

[ref6] Coulton S, Alam MF, Boniface S, et al. (2019) Opportunistic screening for alcohol use problems in adolescents attending emergency departments: an evaluation of screening tools. J Public Health 41:53–60. 10.1093/pubmed/fdy049 PMC6459356 29590416

[ref7] Coulton S, Drummond C, James D, et al. (2006) Opportunistic screening for alcohol use disorders in primary care: comparative study. BMJ 332:511–7. 16488896 10.1136/bmj.38743.421574.7C PMC1388125

[ref8] Coulton S, Watson J, Bland M, et al. (2008) The effectiveness and cost-effectiveness of opportunistic screening and stepped care interventions for older hazardous alcohol users in primary care (AESOPS) - a randomised control trial protocol. BMC Health Serv Res 8:129. 18549492 10.1186/1472-6963-8-129 PMC2442836

[ref9] Deluca P, Coulton S, Alam MF, et al. (2021) Brief interventions to prevent excessive alcohol use in adolescents at low-risk presenting to emergency departments: three-arm, randomised trial of effectiveness and cost-effectiveness. Int J Drug Policy 93:103–13. 10.1016/j.drugpo.2021.103113 PMC8261826 33487528

[ref10] Deluca P, Coulton S, Alam MF, et al. (2020) Screening and brief interventions for adolescent alcohol use disorders presenting through emergency departments: a research programme including two RCTs. Southampton (UK) NIHR Journal Library. 32011840

[ref11] Department for Education . (2017) Permanent and Fixed-period Exclusions in England: 2015 to 2016. London Department for Education.

[ref12] Department of Health & Social Care . (2016) National Health Service Reference Costs 2015–16. London Dept Health & Social Care.

[ref13] Dienes Z, Coulton S, Heather N. (2018) Using Bayes factors to evaluate evidence for no effect: examples from the SIPS project. Addiction 113:240–6. 28804980 10.1111/add.14002

[ref14] Donaldson L . (2009) Guidance on the consumption of alcohol by children and young people. London Dept of Health.

[ref15] Donoghue K, Rose H, Boniface S, et al. (2017) Alcohol consumption, early-onset drinking, and health-related consequences in adolescents presenting at emergency departments in England. J Adolesc Health 60:438–46. 28110867 10.1016/j.jadohealth.2016.11.017

[ref16] Drummond C, Deluca P, Coulton S, et al. (2014) The effectiveness of alcohol screening and brief intervention in emergency departments: a multicentre pragmatic cluster randomized controlled trial. Plos One 9:e99463. 24963731 10.1371/journal.pone.0099463 PMC4070907

[ref17] Emerson E, Robertson J, Baines S, et al. (2016) Predictors of self-reported alcohol use and attitudes toward alcohol among 11-year-old British children with and without intellectual disability. J Intellect Disabil Res 60:1212–26. 27582378 10.1111/jir.12334

[ref18] EuroQol Research . (1990) EQ-5D. Rotterdam EuroQol Research Foundation.

[ref19] Foxcroft DR, Tsertsvadze A. (2011) Universal school-based prevention programs for alcohol misuse in young people. Cochrane Database Syst Rev CD009113. 21901733 10.1002/14651858.CD009308

[ref20] Fuller E . (2015) Smoking, Drinking and Drug Use among Young People in England in 2014. London NatCen Social Research.

[ref21] Giles EL, Coulton S, Deluca P, et al. (2016) A multi-centre individual-randomized controlled trial of screening and brief alcohol intervention to prevent risky drinking in young people aged 14–15 in a high school setting (SIPS JR-HIGH): study protocol. BMJ Open 6:e012474. 10.1136/bmjopen-2016-012474 PMC5223663 28011807

[ref22] Gmel G, Venzin V, Marmet K, et al. (2012) A quasi-randomized group trial of a brief alcohol intervention on risky single occasion drinking among secondary school students. Int J Public Health 57:935–44. 23089675 10.1007/s00038-012-0419-0

[ref23] Hale DR, Fitzgerald-Yau N, Viner RM. (2014) A systematic review of effective interventions for reducing multiple health risk behaviors in adolescence. Am J Public Health 104:19–41. 10.2105/AJPH.2014.301874 PMC3987586 24625172

[ref24] Harbke CR, Laurent J, Catanzaro SJ. (2019) Comparison of the original and short form drinking motives questionnaire-revised with high school and underage college student drinkers. Assessment 26:1179–93. 28938864 10.1177/1073191117731812

[ref25] Kaner E, Bland M, Cassidy P, et al. (2013) Effectiveness of screening and brief alcohol intervention in primary care (SIPS trial): pragmatic cluster randomised controlled trial. BMJ 346:e8501. 23303891 10.1136/bmj.e8501 PMC3541471

[ref26] Kaner EF, Beyer FR, Muirhead C, et al. (2018) Effectiveness of brief alcohol interventions in primary care populations. Cochrane Database Syst Rev 2:CD004148. 29476653 10.1002/14651858.CD004148.pub4 PMC6491186

[ref27] Leigh BC, Gillmore MR, Morrison DM. (1998) Comparison of diary and retrospective measures for recording alcohol consumption and sexual activity. J Clin Epidemiol 51:119–27. 9474072 10.1016/s0895-4356(97)00262-x

[ref28] Lintonen T, Ahlström S, Metso L. (2004) The reliability of self-reported drinking in adolescence. Alcohol Alcohol 39:362–8. 15208172 10.1093/alcalc/agh071

[ref29] Martínez Martínez KI, Pedroza Cabrera FJ, de los Ángeles Vacío Muro M, et al. (2008) Consejo breve Para adolescentes escolares que abusan del alcohol [school-based brief counseling for teenage drinkers]. Acta Ortop Mex 34:247–64.

[ref30] Milner W, Rollnick S. (2013) Motivational Interviewing: Helping People Change. New York Guilford Press.

[ref31] National Institute for Health & Social Care Excellence . (2010) Alcohol-use Disorders–Preventing the Development of Hazardous and Harmful Drinking. London NICE.

[ref32] Newbury-Birch D, Gilvarry E, McArdle P, et al. (2009) The Impact of Alcohol Consumption on Young People: A Review of Reviews. London Department for Children, Schools and Families.

[ref33] Newbury-Birch D, Scott S, O’Donnell A, et al. (2014) A pilot feasibility cluster randomised controlled trial of screening and brief alcohol intervention to prevent hazardous drinking in young people aged 14–15 in a high school setting (SIPS JR-HIGH). Public Health Res. NIHR Journal Library, Southampton, UK. 25642576

[ref34] NHS Digital . (2018) Smoking, Drinking and Drug Use Among Young People in England in 2016. Leeds NHS Digital.

[ref35] NHS Digital . (2016a) Health Survey for England. Leeds NHS Digital.

[ref36] NHS Digital . (2016b) Smoking, Drinking and Drug Use Among Young People. Leeds NHS Digital.

[ref37] O’Neil S, Coulton S, Deluca P, et al. (2012) Brief intervention to prevent hazardous drinking in young people aged 14–15 in a high school setting (SIPS JR-HIGH): study protocol for a randomized controlled trial. Trials 13:166. 22974108 10.1186/1745-6215-13-166 PMC3707809

[ref38] Percy A, McAlister S, Higgins K, et al. (2005) Response consistency in young adolescents’ drug use self-reports: a recanting rate analysis. Addiction 100:189–96. 15679748 10.1111/j.1360-0443.2004.00943.x

[ref39] Personal Social Services Research . (2015) Unit Costs of Health and Social Care 2015. Canterbury PSSRU, University of Kent.

[ref40] Saunders JB, Aasland OG, Babor TF, et al. (1993) Development of the alcohol use disorders identification test (AUDIT): WHO collaborative project on early detection of persons with harmful alcohol consumption–II. Addiction 88:791–804. 8329970 10.1111/j.1360-0443.1993.tb02093.x

[ref41] Shillington AM, Roesch SC, Reed MB, et al. (2011) Typologies of recanting of lifetime cigarette, alcohol and marijuana use during a six-year longitudinal panel study. Drug Alcohol Depend 118:134–40. 21524861 10.1016/j.drugalcdep.2011.03.009 PMC3164929

[ref42] Shono Y, Ames SL, Edwards MC, et al. (2018) The Rutgers alcohol problem index for adolescent alcohol and drug problems: a comprehensive modern psychometric study. J Stud Alcohol Drugs 79:658–63. 30079883 10.15288/jsad.2018.79.658 PMC6090099

[ref43] Sobell L, Sobell M. (1995) Alcohol Timeline Followback Users’ Manual. Toronto, ON Addiction Research Foundation.

[ref44] Strom HK, Adolfsen F, Fossum S, et al. (2014) Effectiveness of school-based preventive interventions on adolescent alcohol use: a meta-analysis of randomized controlled trials. Subst Abuse Treat Prev Policy 9:48. 25495012 10.1186/1747-597X-9-48 PMC4274678

[ref45] Sumnall H, Agus A, Cole J, et al. (2017) Steps towards alcohol misuse prevention programme (STAMPP): a school- and community-based cluster randomised controlled trial. Public Health Res 5:1–154. 28406601

[ref46] Toumbourou JW, Gregg ME, Shortt AL, et al. (2013) Reduction of adolescent alcohol use through family-school intervention: a randomized trial. J Adolesc Health 53:778–84. 23968880 10.1016/j.jadohealth.2013.07.005

[ref47] Viner RM, Taylor B. (2007) Adult outcomes of binge drinking in adolescence: findings from a UK national birth cohort. J Epidemiol Community Health 61:902–7. 17873228 10.1136/jech.2005.038117 PMC2652971

[ref48] Williams R, Vinson DC. (2001) Validation of a single screening question for problem drinking. J Fam Pract 50:307–12. 11300981

[ref49] Wilson DB, Gottfredson DC, Najaka SS. (2001) School-based prevention of problem behaviors: a meta-analysis. J Quantitative Criminol 17:247–72.

[ref50] Winters KC, Leitten W. (2007) Brief intervention for drug-abusing adolescents in a school setting. Psychol Addict Behav 21:249–54. 17563146 10.1037/0893-164X.21.2.249

